# Association of Osteoporosis with Tooth Loss and Dental Radiomorphometric Indices

**DOI:** 10.3390/biomedicines12122886

**Published:** 2024-12-18

**Authors:** Anna Damanaki, Marie Luis Habel, James Deschner

**Affiliations:** Department of Periodontology and Operative Dentistry, University Medical Center, University of Mainz, 55131 Mainz, Germany; mahabel@uni-mainz.de (M.L.H.); james.deschner@uni-mainz.de (J.D.)

**Keywords:** osteoporosis, periodontitis, alveolar bone, tooth loss, dental panoramic radiograph

## Abstract

**Background/Objectives:** Osteoporosis is a systemic disease associated with reduced bone mass, impaired bone microarchitecture, and thus an increased risk of bone fractures. Moreover, patients with osteoporosis are more likely to experience periodontal diseases and tooth loss. Some indices have been proposed to detect osteoporosis on dental panoramic radiographs. The aim of our retrospective study was to investigate the association between osteoporosis and the loss of alveolar bone and teeth and to evaluate the validity of several dental radiomorphometric indices for assessing osteoporosis. **Methods:** In patients with and without osteoporosis, tooth loss, alveolar bone loss, the panoramic mandibular index (PMI), mental index (MI), and mandibular cortical index (MCI) were determined. **Results:** Compared with the non-osteoporotic group, patients with osteoporosis showed more tooth loss and more severe alveolar bone loss. PMI and MI were lower in patients with osteoporosis than in the non-osteoporotic group. Analysis of MCI showed that category C3 (cortical layer forms strong endosteal cortical residues and is clearly porous) was significantly more common in patients with osteoporosis. **Conclusions:** Osteoporosis is associated with more tooth and alveolar bone loss. Furthermore, various dental radiomorphometric indices are altered in osteoporosis and could thus help to better assess osteoporosis of the jaw.

## 1. Introduction

Osteoporosis is a systemic skeletal disorder that predominantly affects adults and is characterized by a decrease in bone density and deterioration of bone microarchitecture, resulting in increased bone fragility [1,2,3]. The prevalence of the disease is 18.3% of the global population, and it is more common in women than in men [4]. The word “osteoporosis” literally means porous bone, indicating low bone density [1].

Osteoporosis is classified into primary and secondary osteoporosis. Primary osteoporosis includes idiopathic, postmenopausal, and senile osteoporosis. Idiopathic osteoporosis is a rare disease that affects young, healthy individuals without any identifiable causes of the disease. Women are particularly affected by osteoporosis due to menopause and the associated reduction in estrogen levels since estrogen has bone-anabolic and anti-inflammatory effects and inhibits osteoclast activity. In addition, normal aging can also cause a decrease in bone density and microarchitecture. Senile osteoporosis is caused by hormonal changes, lack of exercise, poor diet, smoking, alcohol, and other risk factors. Secondary osteoporosis is caused by diseases, e.g., endocrine, gastrointestinal, rheumatic, renal, neurological, hematologic, and genetic diseases, and various medications [5,6,7,8].

Apart from a medical history, the diagnosis of osteoporosis is made radiologically by quantifying bone mineral density using dual-energy X-ray absorptiometry (DEXA) of the hip and spine. DEXA can be used to determine the T-score, which indicates how much the measured bone density differs from the bone density of young, healthy adults. A T-score of <−2.5 means that osteoporosis is present [5,7]. Quantitative computed tomography is also available as a diagnostic tool [9,10]. The Fracture Risk Assessment Tool (FRAX) can be used to determine the need for treatment. FRAX can be used to calculate 10-year fracture risk and decide whether therapeutic intervention is needed [11,12].

There are various therapy options for osteoporosis. These include, for example, taking vitamin D and calcium supplements. Furthermore, antiresorptive drugs, which are taken orally or injected, such as bisphosphonates and anti-RANKL antibodies, play an increasingly important role [13,14,15].

Due to the high prevalence of osteoporosis, it is very often that dentists are confronted with the problems associated with this disease. Patients with osteoporosis often consult a dentist before starting treatment with antiresorptive or anabolic agents to avoid the risk of developing medication-related osteonecrosis of the jaw (MRONJ) due to dental or periodontal infections or subsequent invasive treatments [16,17]. There is also evidence that patients with osteoporosis may have more severe forms of periapical lesions due to reduced jaw bone density and that their healing after endodontic treatment may be compromised [18,19]. Osteoporosis can also increase the risk of dental implant loss or failure [20,21]. It has also been reported that osteoporosis is associated with periodontitis and tooth loss [22,23]. While there are shared risk factors, such as age and smoking, that contribute to this association, evidence suggests that bidirectional causality also exists between osteoporosis and periodontitis as well as tooth loss [22,23,24,25,26,27,28].

In dentistry, X-rays of the teeth and surrounding bone are commonly taken. A dental panoramic radiograph is one of the most common intraoral radiographs and shows the teeth, the bone of the upper and lower jaw, the paranasal sinuses, the temporomandibular joint, and several other structures [29]. Such radiographs are often required before, during, and/or after dental treatment. However, the question is whether such dental X-rays could also provide information about whether osteoporosis is present or how severely the jawbone is affected by osteoporosis. Dental X-rays could thus help to better predict the success of dental therapy and possibly even identify previously undetected osteoporosis as an incidental finding. To date, several markers have been used to detect possible osteoporosis of the jaw. These include the panoramic mandibular index (PMI), mental index (MI), and mandibular cortical index (MCI) [30,31]. Numerous studies have demonstrated that PMI and MI are useful markers for the detection of osteoporosis in panoramic radiographs [30,31,32,33,34], while others have shown that these indices are not reliable enough and do not allow significant differentiation between patients with osteoporosis and healthy individuals [35,36,37,38]. Furthermore, a number of studies including both females and males in whom the presence of osteoporosis was confirmed by measuring bone mass have shown that the MCI could be a strong indicator of osteopenia or osteoporosis [32,34,37,39,40,41,42,43,44].

Although some publications have already been published on this topic, the question still arises as to how valid these indices are, how practical it is to collect them in dental practice, and which of the above indices is best for assessing osteoporosis. Furthermore, cutoff values were given in the literature for some of these indices, although it is unclear whether these cutoff values determined in the studies fit the authors’ own patient cohort. For example, some studies only included postmenopausal women, but not men. In addition, ethnic differences regarding such cutoff values could also play a role. Moreover, a new classification for periodontal and peri-implant diseases and conditions has been in place since 2017, in which certain threshold values for alveolar bone loss and the number of teeth lost due to periodontitis are defined for the staging [45]. However, it is unknown whether the previously reported association between periodontitis and osteoporosis can also be observed when using these threshold values. Therefore, the aim of this retrospective study was to investigate the association between osteoporosis and the loss of alveolar bone and teeth using these thresholds and to evaluate the validity of several dental radiomorphometric indices for assessing jaw osteoporosis.

## 2. Materials and Methods

### 2.1. Osteoporosis Group

Local ethics committee approval (Ethics Committee of the State Medical Association of Rhineland-Palatinate; approval number: 2022-26521) was obtained for this retrospective study.

All records of patients examined and treated in the Department of Periodontology and Operative Dentistry of the University Medical Center of the University of Mainz, Germany, were retrospectively reviewed for the period of 2013–2023. A total of approximately 15,000 records were examined, and all patients with a history of self-reported osteoporosis were included in a registry. A total of 93 patients were identified. For these patients, it was assessed whether a dental panoramic radiograph was available in the radiological records. It was also assessed whether the dental panoramic radiograph coincided in time with the report of the disease. If more than one dental panoramic radiograph was available, the one taken at the time closest to the disease report was selected for further evaluation. Patients were excluded from the registry if no dental panoramic radiograph was available. The next step was to determine whether the dental panoramic radiograph had been taken in the department or in an outpatient clinic and subsequently inserted into the department’s X-ray program. All patients with outpatient radiographs were excluded from the registry, as this would not ensure a standardized interpretation of the radiographs.

### 2.2. Non-Osteoporotic Group

Before the dental panoramic radiographs were analyzed, a non-osteoporotic group was selected from the archive of patient records. This group included the same number of participants as the osteoporosis group. The individuals were matched according to sex and age. Furthermore, it was ensured that these participants had no diseases or medication that could cause osteoporosis. It was also verified that a dental panoramic radiograph was available. A total of 39 patients in the osteoporosis group could be matched with 39 individuals in the non-osteoporotic group.

### 2.3. Study Parameters

The following radiographic parameters were evaluated:

1. The number of missing teeth was determined on each radiograph. Wisdom teeth were not considered. In addition, the number of teeth was assessed on the basis of the classification of periodontal and peri-implant diseases and conditions of the European Federation of Periodontology [45]. Three categories were defined: (1) no tooth loss, (2) a maximum of 4 missing teeth, and (3) 5 or more missing teeth.
2. The maximum alveolar bone loss was measured for each radiograph. Adapted from the classification of periodontal and peri-implant diseases and conditions of the European Federation of Periodontology [45], alveolar bone loss was divided into 2 categories: (1) alveolar bone loss ≤ 33% and (2) alveolar bone loss > 33%.
3. The mental index (MI), also known as mandibular cortical width, was determined as the width of the mandibular cortical bone at the mental foramen.
4. The panoramic mandibular index (PMI) was calculated as the ratio of the cortical width at the mental foramen to the distance between the inferior cortical margin and the inferior margin of the mental foramen.
5. The mandibular cortical index (MCI), also known as the Klemetti index and consisting of three categories, was analyzed [46]:

These three categories were as follows:

C1: The endosteal margin of the cortex is even and sharp on both sides.
C2: The endosteal margin shows semilunar defects (lacunar resorption) and/or appears to form endosteal cortical residues on one or both sides.
C3: The cortical layer forms strong endosteal cortical residues and is clearly porous.

The PMI, MI, and MCI were measured for both the right and left sides of the mandible. For the PMI and MI, the mean value of both sides was used. If one side of the mandible could not be evaluated, the value of the other side was used. If both sides could not be assessed, this was marked as a missing value. For the MCI, the highest category was taken. Again, the category of the other side was used if one side of the mandible could not be scored. If both sides could not be evaluated, this was marked as a missing value.

### 2.4. Software and Analysis of Dental Panoramic Radiographs

The radiologic analysis of the dental panoramic radiographs was performed with the program Sidexis XG (neXt Generation; version 2.63 2016; Dentsply Sirona, Bensheim, Germany) in a darkroom. To ensure quality, the contrast and brightness of the monitor were adjusted using a monitor test image according to DIN 6868-157 TG18-OIQ.

To reduce the influence of subjective assessment, the examiner was calibrated by a second examiner using 30 radiographs prior to the beginning of the evaluation. All radiographs were scored by the same examiner. In cases of ambiguity, the results were discussed to arrive at a joint assessment that was as objective as possible.

### 2.5. Statistics

Statistics were performed using IBM SPSS Statistics software (version 27, IBM SPSS, Chicago, IL, USA). The mean and standard error of the mean were calculated, and homogeneity of variance and normal distribution were examined. The Mann–Whitney U and T-tests were applied to detect statistically significant differences between groups (*p* < 0.05). Further analysis was performed by using the chi-squared test with Bonferroni post hoc. Moreover, crosstabs with pairwise Z-tests output were generated. A receiver operating characteristic curve (ROC curve) was constructed to define data-based cutoff values for PMI and MI.

## 3. Results

### 3.1. Age and Sex

The mean age of the patients in the osteoporosis group was 66.6 years, with a standard deviation of 11.4 years and a range of 29 to 89 years. The mean age of the participants belonging to the non-osteoporotic group was 66.6 ± 11.5 years, with a range of 25 to 85 years. The osteoporosis group comprised 33 females (84.6%) and 6 males (15.4%), yielding a sex ratio of 5.5:1. To ensure a valid comparison, the non-osteoporotic group was matched for sex with the osteoporotic group, resulting in an equal number and proportion of females and males (Table 1).

### 3.2. Osteoporosis and Tooth Loss

**Figure 1:**
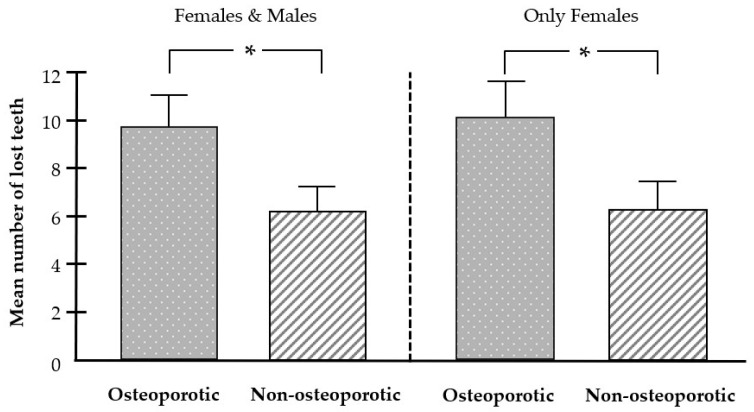
Effect of osteoporosis on tooth loss. Mean number of lost teeth in the osteoporotic (n = 39) and non-osteoporotic (n = 39) groups. Bars show mean ± SEM; * significant (*p* < 0.05) difference between groups.

Patients with osteoporosis showed statistically significantly higher tooth loss compared with the non-osteoporotic group (Figure 1 and Table S1). Even when only females were included in the analysis, tooth loss was again higher in the osteoporosis group than in the non-osteoporotic group (Figure 1).

**Figure 2:**
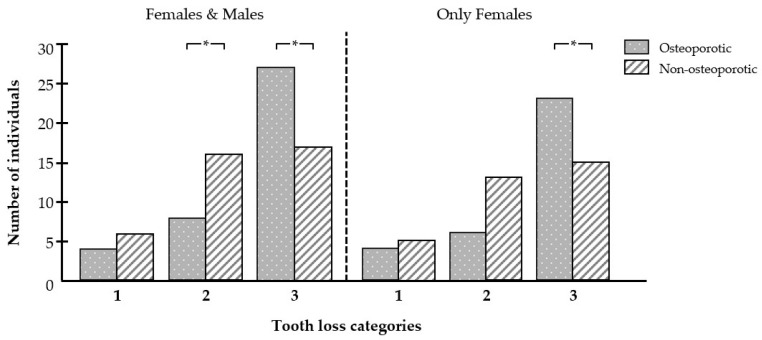
Effect of osteoporosis on the frequency of tooth loss categories. Number of individuals without tooth loss (category 1), with 1 to 4 lost teeth (category 2), and with ≥5 lost teeth (category 3) in the osteoporotic (n = 39) and non-osteoporotic (n = 39) groups. * Significant (*p* < 0.05) difference between groups.

The categorization of tooth loss (number of lost teeth = 0, 1–4, and ≥5) revealed that patients with osteoporosis had lost five or more teeth at a significantly higher rate than individuals without osteoporosis, who had significantly more often lost one to four teeth (Figure 2). When the analysis was limited to female participants, the same pattern was observed: patients with osteoporosis were statistically significantly more likely to have lost five or more teeth than those without osteoporosis (Figure 2).

### 3.3. Osteoporosis and Alveolar Bone Loss

**Figure 3:**
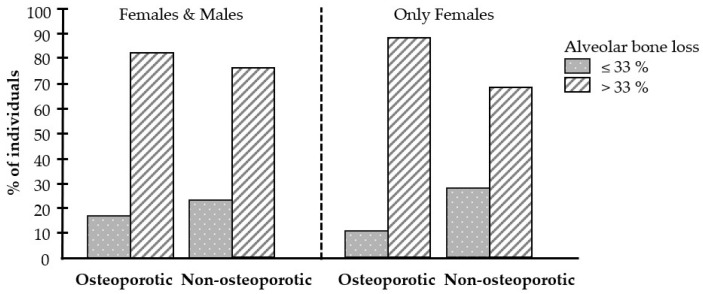
Effect of osteoporosis on alveolar bone loss. Percentage of participants with minor (≤33%) or major (>33%) alveolar bone loss in the osteoporotic and non-osteoporotic groups.

Individuals in the osteoporotic and non-osteoporotic groups with alveolar bone loss were divided according to the extent (≤33% and >33%) of the bone loss. While 82.9% of the osteoporotic group showed alveolar bone loss of >33%, 76.5% of the non-osteoporotic group had this high level of alveolar bone loss (Figure 3). When analyzing females only, the difference was even more pronounced; 89.7% of females with osteoporosis demonstrated an alveolar bone loss of >33%, compared with only 71.4% of females without osteoporosis (Figure 3).

### 3.4. Osteoporosis and Panoramic Mandibular Index (PMI)

**Figure 4:**
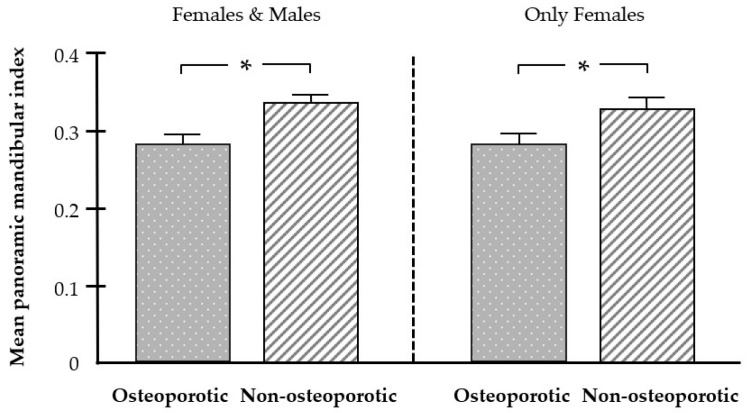
Effect of osteoporosis on the panoramic mandibular index (PMI). Mean PMI in the osteoporotic (n = 39) and non-osteoporotic (n = 39) groups. Bars show mean ± SEM; * significant (*p* < 0.05) difference between groups.

The analysis of the panoramic radiographs revealed that the PMI was significantly lower in patients with osteoporosis than in the non-osteoporotic group. The significantly lower PMI in osteoporosis was also observed when only females were included in the analysis (Figure 4).

To identify patients with osteoporosis, the sensitivity and specificity for various PMI cutoff values between 0.31 and 0.39 were then calculated. When both sexes were included, the sensitivity ranged between 54.8% and 96.8%, and the specificity ranged between 25.0% and 65.5% for these cutoff values. When only panoramic radiographs of females were analyzed, the sensitivity ranged from 53.8% to 96.2%, and the specificity ranged from 19.2% to 61.5% (Table 2).

### 3.5. Osteoporosis and Mental Index (MI)

**Figure 5:**
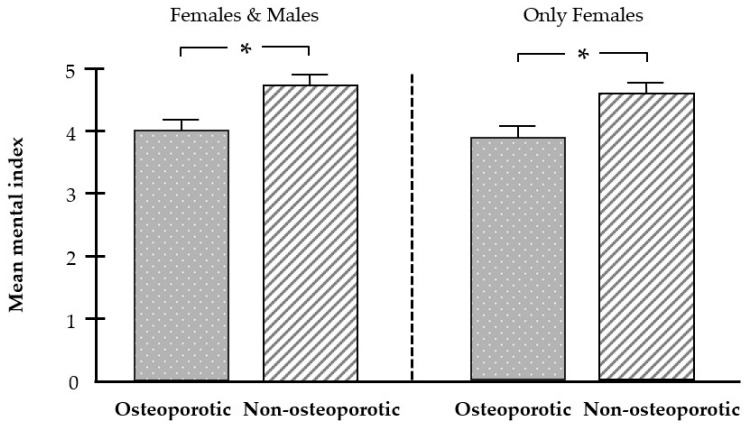
Effect of osteoporosis on the mental index (MI). Mean MI in the osteoporotic (n = 39) and non-osteoporotic (n = 39) groups. Bars show mean ± SEM; * significant (*p* < 0.05) difference between groups.

The radiographic analysis also demonstrated that the MI was significantly lower in patients with osteoporosis than in the non-osteoporotic group. Even when only females were included in the analysis, a significantly lower MI was again observed in osteoporosis (Figure 5).

Sensitivity and specificity were then calculated for different MI cutoff values between 4.2 and 5.0 to distinguish patients with osteoporosis. When including females and males, the sensitivity ranged from 58.1% to 90.3%, and the specificity ranged from 31.3% to 65.6%. When only females’ panoramic radiographs were included in the analysis, the sensitivity ranged from 65.4% to 88.5%, and the specificity ranged from 23.1% to 65.4% (Table 3).

### 3.6. Osteoporosis and Mandibular Cortical Index (MCI)

**Figure 6:**
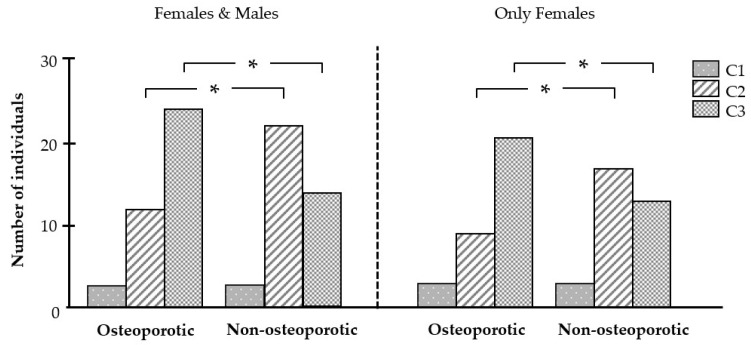
Effect of osteoporosis on the frequency of the mandibular cortical index (MCI) categories. Number of individuals with category C1, C2, and C3, respectively, in the osteoporotic (n = 39) and non-osteoporotic (n = 39) groups. * significant (*p* < 0.05) difference between groups.

Differences between the osteoporotic and non-osteoporotic groups were also observed for the MCI. Although category C1 occurred with similar frequency in both groups, category C2 was found significantly more often in the non-osteoporotic group, and category C3 was significantly more common in the osteoporotic group (Figure 6). The same distribution of categories was also observed when only females were included in the analysis (Figure 6).

When the C3 category of the MCI was used as an indicator of osteoporosis, the sensitivity reached 61.5% and the specificity reached 64.1% (Table 4). When only women were analyzed, the sensitivity amounted to 63.6% and the specificity to 60.6% (Table 4).

## 4. Discussion

The aim of this study was to investigate the association between osteoporosis and the loss of alveolar bone and teeth and to evaluate the validity of several dental radiomorphometric indices for assessing osteoporosis. Compared with individuals without osteoporosis, patients with osteoporosis showed more tooth loss and more severe alveolar bone loss. Furthermore, various dental radiomorphometric indices were altered in osteoporosis and could thus help to better assess osteoporosis of the jaw.

Osteoporosis is characterized by reduced bone density and altered microarchitecture and is more common in women than in men. In our retrospective analysis, the proportion of women was approximately 85%, which confirms that women are at higher risk of osteoporosis, mainly due to hormonal changes during menopause [4,47,48]. Moreover, the mean age of the osteoporosis patients was 67 years, underlining that osteoporosis occurs mainly in later life.

First, we examined the effects of osteoporosis on tooth loss. Our study showed that patients with osteoporosis had more tooth loss than individuals without osteoporosis. We then also examined the effects of osteoporosis on the frequency of tooth loss categories (no tooth loss, one to four lost teeth, and 5 or more lost teeth). These categories of tooth loss were selected according to the classification of periodontal and peri-implant diseases and conditions of the European Federation of Periodontology, as these categories help determine the staging of periodontitis [45]. The categorization of tooth loss revealed that osteoporotic patients had lost 5 or more teeth at a higher rate than individuals without osteoporosis, who had lost one to four teeth significantly more often. In osteoporosis, the jawbone is also affected by systemic bone loss and altered microarchitecture. This makes the maxilla and mandible more susceptible to periodontitis-induced bone destruction, which can lead to increased tooth loss if periodontitis is not successfully treated [27,49]. That osteoporosis is associated with more tooth loss has also been demonstrated by other studies, thus supporting our study [27,49,50,51,52,53]. There is also evidence that no or only short-term treatment of osteoporosis is associated with a fourfold increased risk of tooth loss [54]. Furthermore, individuals with a history of vertebral fractures have a higher risk of tooth loss, which again highlights the link between tooth loss and osteoporosis [55].

Since tooth loss is often the result of periodontitis, it is not surprising that periodontitis has also been reported to be associated with osteoporosis [23,24,54,56]. Periodontitis is a multifactorial, chronic, inflammatory disease of the periodontium, which is characterized, among other features, by alveolar bone loss. Therefore, in our study, the osteoporotic and non-osteoporotic individuals with alveolar bone loss were categorized according to the extent of bone loss (≤33% and >33%). These two categories of alveolar bone loss were again chosen according to the European Federation of Periodontology classification of periodontal and peri-implant diseases and conditions, as both categories are helpful in determining the staging of periodontitis [45]. Our study showed that patients with osteoporosis were more likely to have alveolar bone loss of >33% than osteoporosis-free patients. The difference between the osteoporotic and non-osteoporotic groups was even more pronounced when only women were included in the analysis.

Overall, our study shows that osteoporosis is associated with the loss of teeth and alveolar bone. Numerous pathomechanisms have been described for this association, although a distinction must be made between shared risk factors for osteoporosis and periodontitis/tooth loss and causality, i.e., a cause-and-effect relationship, between osteoporosis and periodontitis/tooth loss. Common risk factors for osteoporosis and periodontitis or tooth loss include older age, smoking, socioeconomic level, unhealthy diet, vitamin D deficiency, bone mineral diseases, and certain medications [22,26,27,57]. However, it is also biologically plausible that there is a causal relationship between osteoporosis and periodontitis/tooth loss, whereby it can be assumed that this causality is bidirectional, i.e., that both conditions have a negative impact on each other. Osteoporosis is a systemic disease that also affects the jawbone. Lower bone density and altered trabecular microarchitecture facilitate microbial-induced alveolar bone loss in periodontitis. As with periodontitis, osteoporosis is associated with a systemic subclinical inflammatory state, so that local periodontal inflammation is further aggravated by osteoporosis [22,26,27,28]. On the other hand, periodontitis could contribute to an increase in osteoporotic bone loss through elevated systemic levels of inflammatory mediators, microorganisms, their components, and metabolic metabolites [22,26,27,28].

Postmenopausal women have an increased risk of osteoporosis because they are subject to aging, as are men, but they also have estrogen deficiency due to menopause. Estrogen has bone-anabolic, anti-inflammatory, and anti-osteoclastic effects. Therefore, alveolar bone loss is increased in postmenopausal women compared with age-matched osteoporosis-free women, younger women who have not yet experienced menopause, and younger adults in general [58,59,60,61,62].

Due to the association of osteoporosis with periodontitis, as well as the loss of alveolar bone and teeth, osteoporosis has great clinical significance in dental practice. The prognosis of teeth and dental implants may be dependent on the presence of osteoporosis. Furthermore, patients with osteoporosis are increasingly receiving antiresorptive or osteoanabolic medications that can lead to the development of MRONJ in the presence of oral infections [63,64]. Therefore, it is helpful if the influence of osteoporosis on the jaw can be assessed with the help of dental radiographs, which are routinely taken in dental practice. Various indices such as the PMI, MI, and MCI have been proposed. Cutoff values have also been reported that should help to identify whether there are osteoporotic changes in the jaw. In this context, the question arises as to how valid such indices are and how practical it is to collect this data in dental practices. The PMI and MI are frequently used in research to assess the presence of osteopenia or osteoporosis in dental panoramic radiographs. The majority of these studies focused on females, especially postmenopausal women [31,32,38,52]. Only a few studies also included males [30,34]. Overall, the results of the various studies are not yet conclusive. Some studies have confirmed the benefit of using these indices to detect osteoporosis [30,31,32,33,34,52], while other studies have found no significant differences between patients with osteoporosis and non-osteoporotic individuals with regard to the PMI and MI [35,36,37,38]. Our analysis of dental panoramic radiographs showed that the PMI and MI were lower in patients with osteoporosis than in the non-osteoporotic group. Even when only women were analyzed, osteoporosis was again associated with a lower PMI and MI. Overall, this showed that both indices are indeed altered in osteoporosis.

In most studies, cutoff values are set for both the PMI and MI. A value below the set cutoff indicates the presence of osteopenia or osteoporosis. The most commonly used cut-off values for the PMI are 0.3 and 0.4 [30,31,65] and 3 mm and 4.3 mm for the MI [31,66]. In this study, we applied a range of cutoff values that were between those previously mentioned. Our results are consistent with the current literature and show that lower PMI cutoff values lead to lower sensitivity and higher specificity. From this, it can be concluded that using low cutoff values is not an effective method for detecting osteoporosis, as it leads to a high number of undiagnosed cases. Conversely, applying higher cutoff values may result in healthy patients being classified as having osteoporosis. With regard to MI, it should be noted that using low cutoff values results in a sensitivity of around 60% with a corresponding specificity. This index is considered to be the most ideal for detecting osteoporosis because it can be easily measured without the need for specialized experience in diagnosing dental panoramic radiographs. Differences between the osteoporotic and non-osteoporotic groups were also found for the MCI. Although category C1 occurred with similar frequency in both groups, category C2 was observed more often in individuals without osteoporosis, whereas category C3 was found more frequently in patients with osteoporosis. Category C3 is characterized by pronounced bone porosity and is considered pathological [39,42,44]. By contrast, category C2, although showing changes in bone structure, is considered a consequence of normal aging [67,68]. Other studies have also shown that category C3 is a reliable indicator of osteopenia or osteoporosis [32,34,37,39,40,41,42,43,44,52]. When category C3 of MCI was used as an indicator for osteoporosis, sensitivity and specificity were around 60% in our study. However, a wide range of sensitivity and specificity values have been reported in the literature [30,31,38,69]. The evaluation of this index requires a specialized examiner, but even in this case, subjectivity remains an issue [65]. Therefore, it is questionable whether this index is suitable for use in daily dental practice.

It should be noted that our study is not without limitations. It is a retrospective analysis and the number of individuals with self-reported osteoporosis and without osteoporosis was limited. Nevertheless, numerous significant differences were observed. Since the proportion of males with and without osteoporosis in our retrospective study was low, no conclusions could be drawn for males alone with regard to the association between osteoporosis and loss of alveolar bone and teeth and radiomorphological indices. There is little evidence of the association between radiomorphometric indices and tooth loss. Tanaka et al. (2020) [53] reported that tooth loss was also significantly correlated with MI and MCI. We were unable to consider this aspect in our study because the sample size of 39 patients per group was not large enough. Further studies are needed to clarify this correlation. It also would have been desirable to have information on bone mineral density/T-score, menopausal status, medication for osteoporosis, and clinical periodontal parameters, as well as reasons for tooth loss. Future studies could contribute to the establishment of new indices or their combinations. It is also conceivable that artificial intelligence could be helpful in identifying osteoporotic changes in the jaw.

## 5. Conclusions

Our study showed that osteoporosis is associated with an increased loss of alveolar bone and teeth. Furthermore, several radiomorphometric indices were altered in osteoporosis. The various dental radiomorphometric indices in osteoporosis could thus be useful in the assessment of osteoporosis of the jaw. The MI proved to be a practical index with acceptable sensitivity and specificity, while the MCI, although more difficult to interpret, may also be useful. Our results emphasize the need to thoroughly screen patients with osteoporosis for periodontal diseases and to treat them if necessary. Furthermore, patients with osteoporosis should be well informed about the increased risk of periodontitis as well as bone and tooth loss.

## Figures and Tables

**Table 1:** Age and sex of the osteoporotic and non-osteoporotic groups.

		Osteoporotic	Non-Osteoporotic
Age	Mean ± Standard Deviation	66.6 ± 11.4	66.6 ± 11.5
	Minimum	29	25
	Maximum	89	85
Sex	Female	33 (84.6%)	33 (84.6%)
	Male	6 (15.4%)	6 (15.4%)

**Table 2:** Sensitivity and specificity of the panoramic mandibular index (PMI) for different cutoff values.

	Cutoff Value	Sensitivity (%)	Specificity (%)
Females and Males	0.31	54.8	65.5
	0.33	74.2	46.9
	0.35	77.4	40.6
	0.37	87.1	37.5
	0.39	96.8	25.0
Only Females	0.31	53.8	61.5
	0.33	73.1	46.2
	0.35	76.9	38.5
	0.37	84.6	34.6
	0.39	96.2	19.2

**Table 3:** Sensitivity and specificity of the mental index (MI) for different cutoff values.

	Cutoff Value	Sensitivity (%)	Specificity (%)
Females and Males	4.2 mm	58.1	65.6
	4.4 mm	61.3	59.4
	4.6 mm	64.5	50.0
	4.8 mm	77.4	40.6
	5.0 mm	90.3	31.3
Only Females	4.2 mm	65.4	65.4
	4.4 mm	65.4	57.7
	4.6 mm	69.2	46.2
	4.8 mm	80.8	34.6
	5.0 mm	88.5	23.1

**Table 4:** Sensitivity and specificity of MCI if category C3 is used as indicator of osteoporosis.

	Category	Sensitivity (%)	Specificity (%)
Females and Males	3	61.5	64.1
Only Females	3	63.6	60.6

## Data Availability

The original contributions presented in this study are included in the article/Supplementary Materials. Further inquiries can be directed to the corresponding author.

## Supplementary Materials

The following supporting information can be downloaded at: https://www.mdpi.com/article/10.3390/biomedicines12122886/s1. Table S1. Tooth loss by age category in patients with osteoporosis and healthy subjects.
