# Supplementary material for: Association of Osteoporosis with Tooth Loss and Dental Radiomorphometric Indices
Source: Biomedicines. 2024 Dec 18;12(12):2886. doi: 10.3390/biomedicines12122886 (PMC11672889; doi:10.3390/biomedicines12122886)
Supplement: Supplementary file 1 [file biomedicines-12-02886-s001.zip › biomedicines-3350685-supplementary.pdf]

Table S1. Tooth loss by age category in patients with osteoporosis and healthy subjects.

Age (Years)	Osteoporotic		Non-Osteoporotic		Osteoporotic & Non-Osteoporotic	
	Mean Number of Lost Teeth	Number of Individuals	Mean Number of Lost Teeth	Number of Individuals	Mean Number of Lost Teeth	Number of Individuals
20-29	0.00	1	0.00	1	0.00	2
30-39	0.00	0	0.00	0	0.00	0
40-49	23.00	1	0.00	1	11.50	2
50-59	8.00	6	6.00	8	6.57	14
60-69	7.86	15	7.00	13	7.46	28
70-79	11.91	11	5.69	13	8.54	24
80-89	11.80	5	9.33	3	10.87	8
